# Supplementary material for: Rapid glacier retreat and downwasting throughout the European Alps in the early 21st century
Source: Nat Commun. 2020 Jun 25;11:3209. doi: 10.1038/s41467-020-16818-0 (PMC7316704; doi:10.1038/s41467-020-16818-0)
Supplement: Supplementary file 1 — Supplementary Information [file 41467_2020_16818_MOESM1_ESM.pdf]

**Supplementary information:**

***Rapid glacier retreat and downwasting throughout the European Alps in the early 21st century***

Sommer et al.

## Supplementary Table 1

Glacier median elevation, elevation range and area per subregion of each glacier inventory (1999-2001, 2011 & 2013-2015) and area change rates (dSdt) over the observation periods (2000 - 2011 & 2000 - 2014). The median acquisition date of the glacier outlines of each subregion is area-weighted by the glacier area enclosed by outlines from the respective dates.

| *ID* | *Region Name* | *Median glacier elevation [m a.s.l.]* | *Glacier elevation range [m a.s.l.]* | *Median Date 1999-2001* | *Area [km²] 1999-2001* | *Median Date 2011* | *Area [km²] 2011* | *Median Date 2013-2015* | *Area [km²] 2013-2015* | *dSdt 2000-2011 [km² a⁻¹]* | *dSdt 2000-2014 [km² a⁻¹]* |
|---|---|---|---|---|---|---|---|---|---|---|---|
| 01 | Dauphiné Alps | 3021 | 2129-4025 | 1999-08-02 | 86.831 | 2011-07-02 | 70.056 | 2014-09-03 | 63.493 | -1.41 ± 0.663 | -1.549 ± 0.478 |
| 02 | Graian Alps | 3074 | 1388-4797 | 2000-08-20 | 340.801 | 2011-07-02 | 266.859 | 2014-09-12 | 241.468 | -6.823 ± 1.707 | -7.074 ± 1.449 |
| 03 | Pennine Alps | 3223 | 1848-4541 | 2000-08-20 | 442.134 | 2011-07-02 | 382.008 | 2014-09-12 | 367.64 | -5.542 ± 1.821 | -5.305 ± 1.44 |
| 04 | Bernese Alps | 2929 | 1303-4140 | 2000-08-20 | 485.754 | 2011-09-13 | 388.275 | 2013-08-01 | 377.319 | -8.823 ± 2.07 | -8.807 ± 1.745 |
| 05 | Glarus Alps | 2736 | 1626-3585 | 1999-09-12 | 53.725 | 2011-09-13 | 38.02 | 2014-07-03 | 36.079 | -1.31 ± 0.283 | -1.193 ± 0.268 |
| 06 | Lepontine Alps | 2841 | 2198-3417 | 1999-09-12 | 54.593 | 2011-09-13 | 37.155 | 2013-08-01 | 35.769 | -1.455 ± 0.347 | -1.357 ± 0.362 |
| 07 | Rhaetian Alps West | 2912 | 2006-3984 | 1999-09-12 | 150.884 | 2011-09-13 | 112.902 | 2014-09-14 | 104.083 | -3.169 ± 0.826 | -3.123 ± 0.714 |
| 08 | Rhaetian Alps East | 3049 | 2106-3736 | 2000-08-22 | 220.91 | 2011-08-21 | 179.768 | 2015-09-01 | 166.016 | -3.747 ± 1.074 | -3.658 ± 0.81 |
| 09 | Rhaetian Alps South | 3089 | 2305-3872 | 2000-08-22 | 126.568 | 2011-08-21 | 103.418 | 2014-09-14 | 97.829 | -2.108 ± 0.664 | -2.047 ± 0.528 |
| 10 | Tauern Alps West | 2895 | 1884-3635 | 1999-09-14 | 211.163 | 2011-08-14 | 164.074 | 2013-09-04 | 157.289 | -3.957 ± 1.072 | -3.861 ± 0.941 |
| | | | | | | | | | | | |
| *I* | Western Alps | 3039 | 1303-4797 | 2000-08-20 | 1474.443 | 2011-09-11 | 1187.335 | 2014-09-12 | 1126.232 | -25.999 ± 7.025 | -25.184 ± 5.715 |
| *II* | Eastern Alps | 2983 | 1843-3984 | 1999-09-14 | 718.831 | 2011-08-21 | 566.474 | 2014-09-14 | 531.468 | -12.784 ± 3.566 | -12.507 ± 2.953 |
| ***Sum/Area weighted date*** | total | | | 2000-08-20 | 2193.274 | 2011-08-23 | 1753.809 | 2014-09-12 | 1657.7 | -39.991 ± 10.925 | -38.527 ± 8.865 |

## Supplementary Figure 1

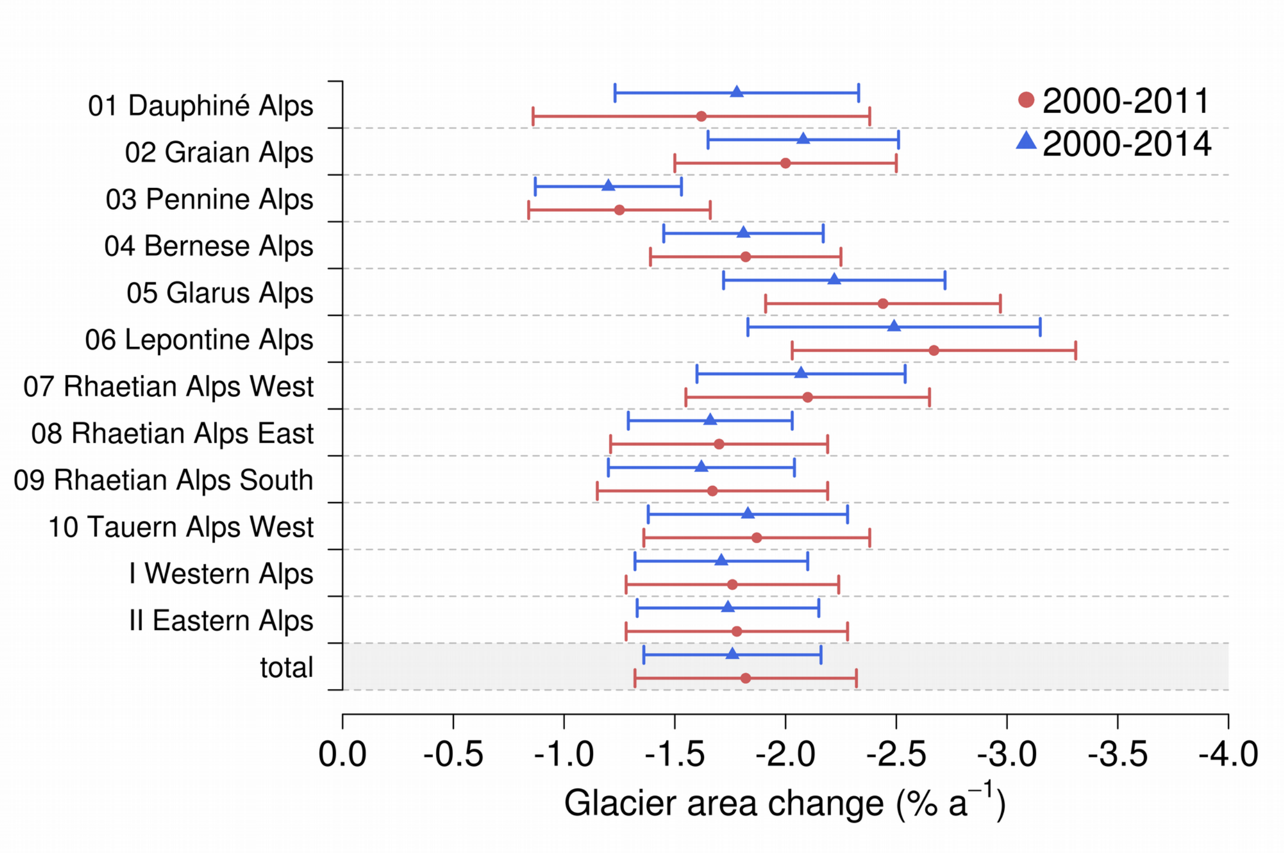


**Supplementary Fig. 1:** Glacier area change rates of all subregions in percent during 2000 - 2011 (red dots) and 2000 - 2014 (blue triangles) and respective uncertainties.

**Supplementary Figure 2**

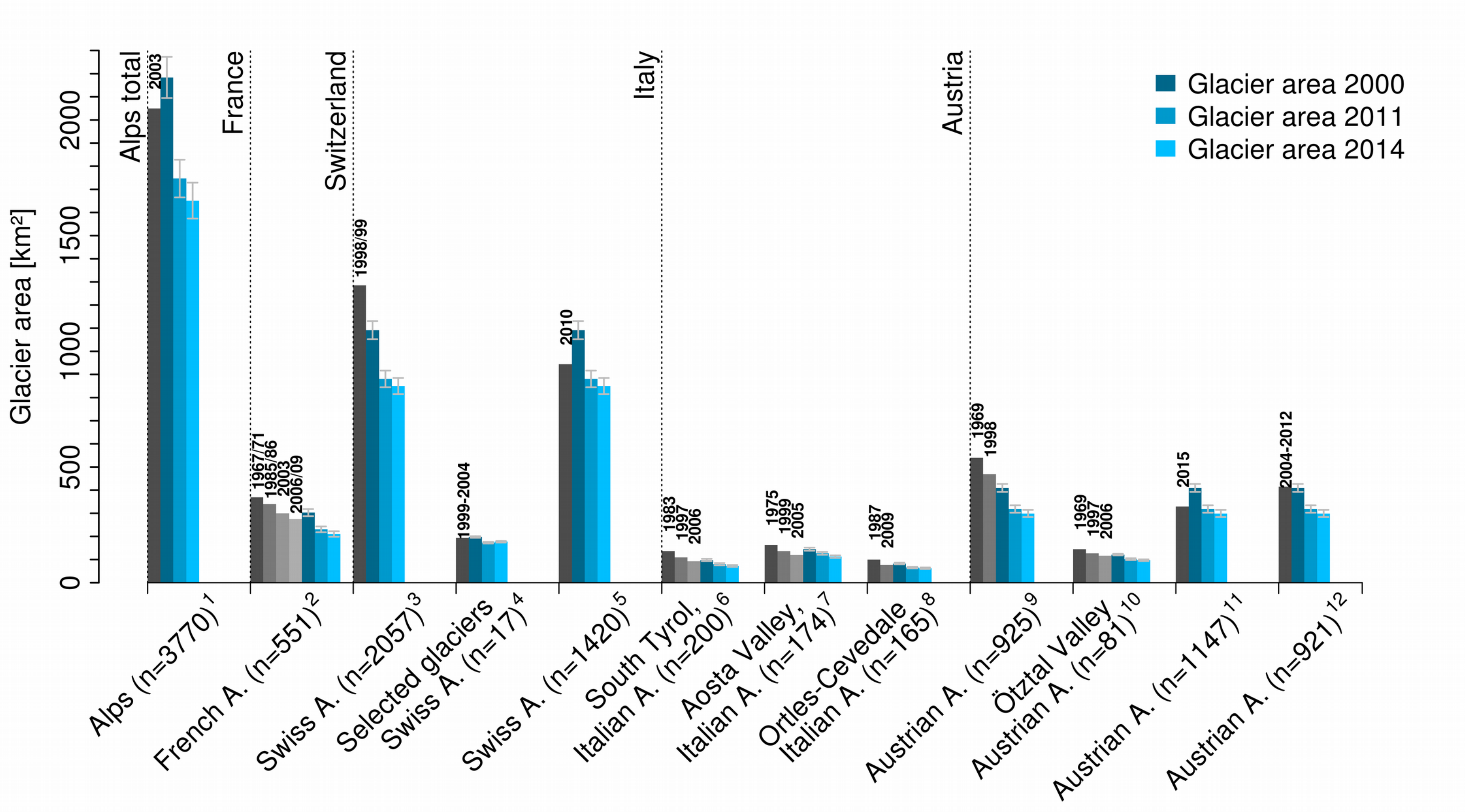


**Supplementary Fig. 2:** Glacier area measurements of different time periods and glacier regions in the European Alps (black to gray bars) grouped by country and compared to respective glacier areas (blue bars) and area uncertainties (gray error bars) of this study (2000, 2011 & 2014). Numbers of glaciers, region and acquisition year of reference areas are given in brackets and above area bars. Uncertainties of glacier areas of this study are based on an empirical area determination error scaled by the respective regional glacier perimeter to area ratio (see methods section).

## Supplementary Figure 3

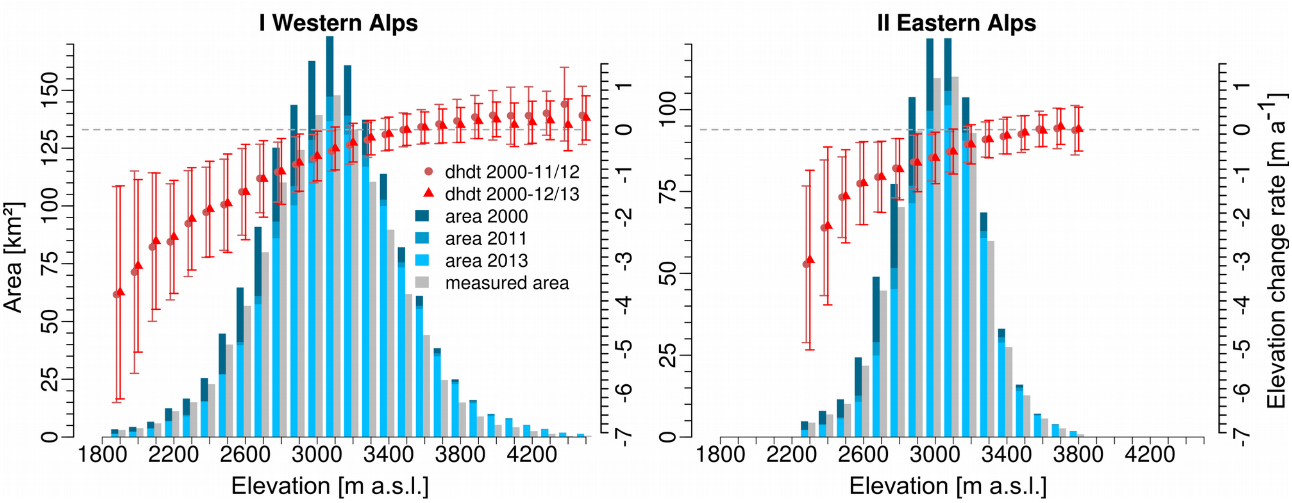


**Supplementary Fig. 3:** Hypsometric distribution of average glacier elevation change rates of 2000-2012 (dark red dots) and 2000-2014 (red triangles) and respective normalized median absolute deviations (error bars). Glacier areas of each elevation bin are shown as blue superimposed bars, gray bars indicate the average glacier area measured by TanDEM-X and SRTM (between 2000-2012 & 2000-2014) according to the 2000 glacier area. Elevation bins with less than 2 km² of glacier area are not shown.

**Supplementary Table 2**

Average glacier-specific mass change rates from glaciological (1999/00-2010/11 & 1999/00-2012/13)[13] and geodetic measurements (2000-2012 & 2000-2014) and regional geodetic mass changes of the respective subregions.

| WGMS ID | Glacier Name | Region Name | Mean glaciological mass change [m.w.e. $a^{-1}$] 1999/00-2010/11 | Mean glacier geodetic mass change [m.w.e. $a^{-1}$] 2000-2012 | Mean glaciological mass change [m.w.e. $a^{-1}$] 1999/00-2012/13 | Mean glacier geodetic mass change [m.w.e. $a^{-1}$] 2000-2014 | Mean regional geodetic mass change [m.w.e. $a^{-1}$] 2000-2012 | Mean regional geodetic mass change [m.w.e. $a^{-1}$] 2000-2014 |
|---|---|---|---|---|---|---|---|---|
| 356 | Saint Sorlin | | -1.837 | -1.244 ± 0.151 | -1.798 | -1.694 ± 0.135 | | |
| 357 | Sarennes | Dauphine A. | -2.351 | -2.577 ± 0.392 | -2.308 | -2.235 ± 0.368 | -0.690 ± 0.266 | -0.739 ± 0.237 |
| | | | | | | | | |
| 354 | Argentiere | | -1.243 | -1.025 ± 0.081 | -1.213 | -0.744 ± 0.070 | | |
| 1264 | Ciardoney | | -1.409 | -0.890 ± 0.225 | -1.411 | -0.790 ± 0.198 | | |
| 352 | Gebroulaz | Graian A. | -0.848 | -0.777 ± 0.134 | -0.858 | -0.684 ± 0.113 | -0.723 ± 0.141 | -0.604 ± 0.116 |
| | | | | | | | | |
| 394 | Allalin | | -0.396 | -0.394 ± 0.163 | -0.417 | -0.434 ± 0.141 | | |
| 366 | Corbassiere | | -0.774 | -0.691 ± 0.125 | -0.799 | -0.607 ± 0.112 | | |
| 367 | Gietro | | -0.639 | -0.626 ± 0.135 | -0.648 | -0.623 ± 0.118 | | |
| 3332 | Hohlaub | | -0.577 | -0.843 ± 0.177 | -0.586 | -0.744 ± 0.151 | | |
| 395 | Schwarzberg | Pennine A. | -0.707 | -0.682 ± 0.107 | -0.685 | -0.588 ± 0.090 | -0.544 ± 0.128 | -0.562 ± 0.114 |
| | | | | | | | | |
| 2660 | Claridenfirn | Glarus A. | -0.778 | -0.670 ± 0.213 | -0.805 | -0.709 ± 0.183 | -0.985 ± 0.211 | -1.031 ± 0.208 |
| | | | | | | | | |
| 463 | Basodino | | -0.824 | -0.704 ± 0.189 | -0.800 | -0.736 ± 0.162 | | |
| 359 | Gries | Lepontine A. | -1.323 | -1.151 ± 0.182 | -1.319 | -1.123 ± 0.160 | -0.974 ± 0.233 | -0.889 ± 0.204 |
| | | | | | | | | |
| 480 | Jamtal F. | | -0.893 | -0.864 ± 0.117 | -0.886 | -0.916 ± 0.104 | | |
| 408 | Silvretta | Raethian A. west | -0.713 | -1.111 ± 0.109 | -0.729 | -0.802 ± 0.097 | -0.809 ± 0.176 | -0.841 ± 0.167 |
| | | | | | | | | |
| 491 | Hintereis F. | | -1.064 | -0.957 ± 0.151 | -1.060 | -1.058 ± 0.132 | | |
| 507 | Kesselwand F. | | -0.353 | -0.312 ± 0.207 | -0.352 | -0.365 ± 0.183 | | |
| 675 | Pendente (Vedr.) | | -1.141 | -1.395 ± 0.118 | -1.171 | -1.243 ± 0.107 | | |
| 489 | Vernagt F. | Raethian A. east | -0.710 | -0.804 ± 0.192 | -0.721 | -0.805 ± 0.171 | -0.739 ± 0.147 | -0.770 ± 0.134 |
| | | | | | | | | |
| 635 | Careser | | -1.731 | -1.683 ± 0.185 | -1.733 | -1.789 ± 0.187 | | |
| 1507 | Fontana Bianca | Raethian A. south | -1.056 | -0.680 ± 0.285 | -1.047 | -1.596 ± 0.246 | -0.831 ± 0.152 | -0.814 ± 0.138 |
| | | | | | | | | |
| 1305 | Goldberg K. | | -0.779 | -0.558 ± 0.144 | -0.815 | -0.632 ± 0.135 | | |
| 547 | Kleinfleiss K. | | -0.587 | -0.605 ± 0.197 | -0.603 | -0.320 ± 0.178 | | |
| 573 | Stubacher Sonnblick K. | | -0.900 | -0.465 ± 0.161 | -0.867 | -0.973 ± 0.149 | | |
| 545 | Wurten K. | Tauern A. west | -0.913 | -1.769 ± 0.186 | -0.944 | -1.011 ± 0.166 | -0.660 ± 0.185 | -0.635 ± 0.168 |

**Supplementary Figure 4**

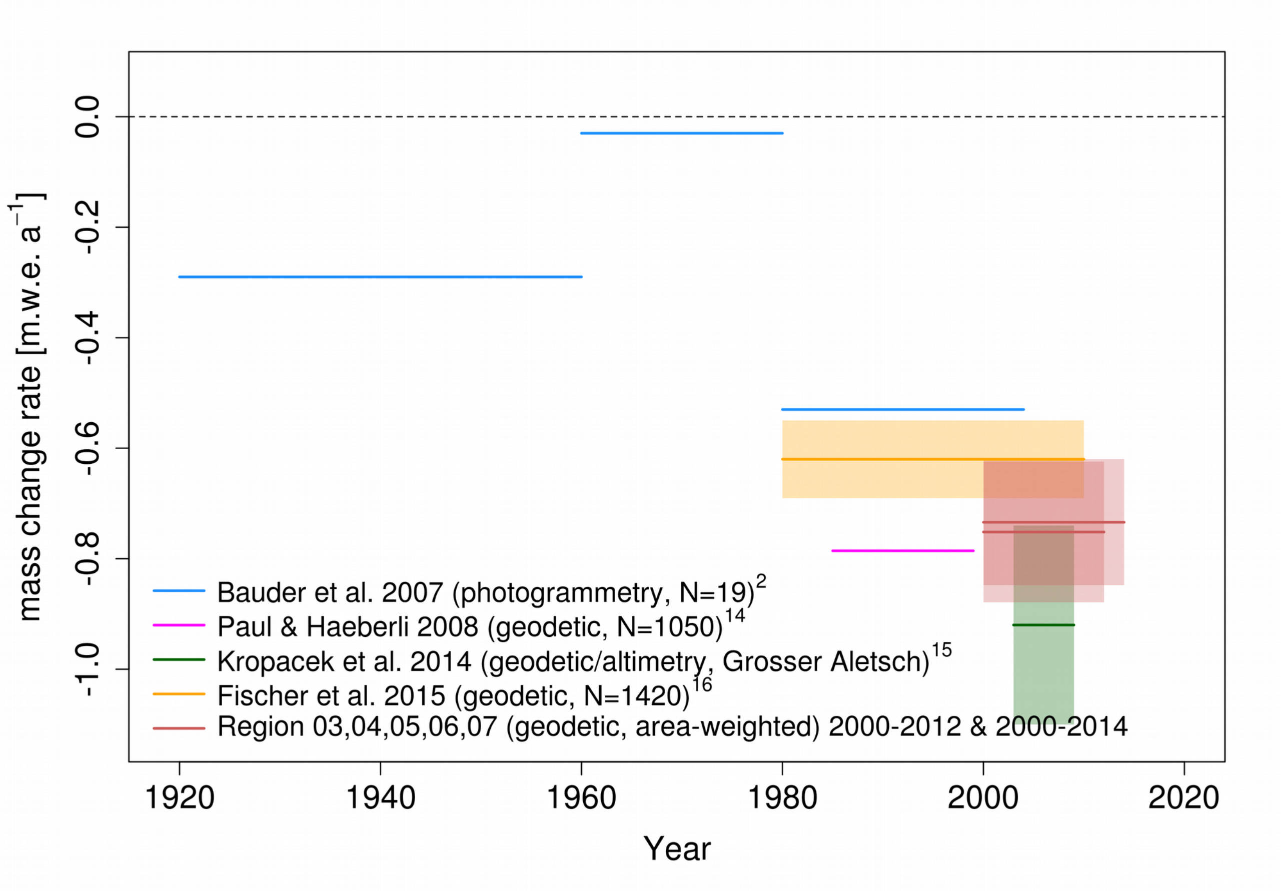


**Supplementary Fig. 4:** Geodetic mass change measurements of selected Swiss glaciers. Fischer et al. (2015)[16] cover all Swiss glaciers according to the Swiss SGI2010 inventory. TanDEM-X-SRTM mass changes are derived from area-weighted values of regions 03-07, including some glaciers in Italy and Austria. Semitransparent error boxes of this study refer to regional mass change uncertainties (see methods section).

## Supplementary Figure 5

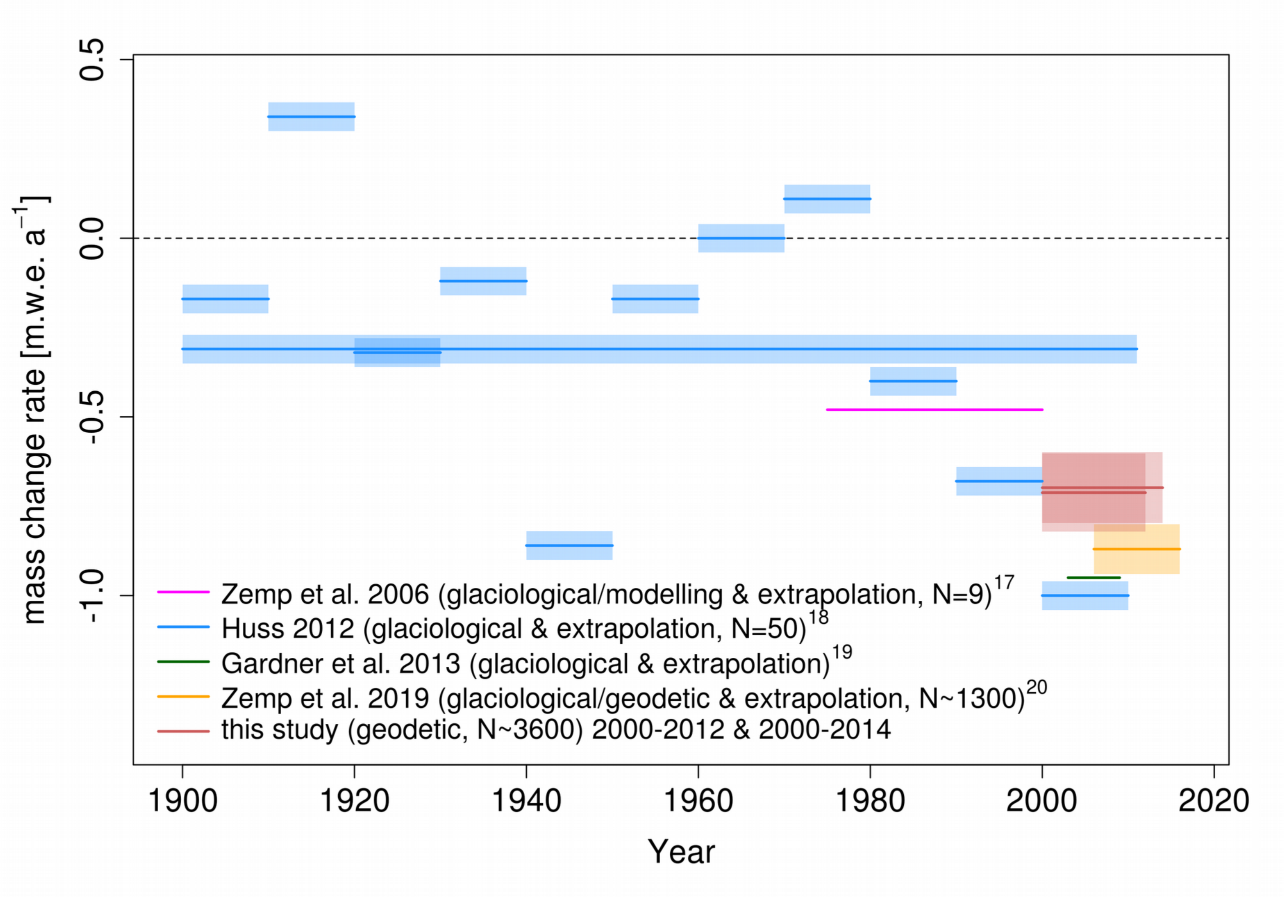


**Supplementary Fig. 5:** Mass change estimates of the entire Alps extrapolated from glaciological and geodetic measurements and modeling. Applied approach and number of measured glaciers (if provided) are given in brackets. Zemp et al. 2006[17] used the mean mass change of nine glaciers to estimate the cumulative change for an extrapolated mean glacier area between 1975-2000 of the entire Alps. Specific mass changes of Gardner et al. (2013)[19] and Zemp et al. (2019)[20] refer to Randolph Glacier Inventory region 11 Central Europe, including glaciers outside the Alps. Semitransparent error boxes of this study refer to alpine-wide mass change uncertainties (see methods section).

**Supplementary Figure 6**

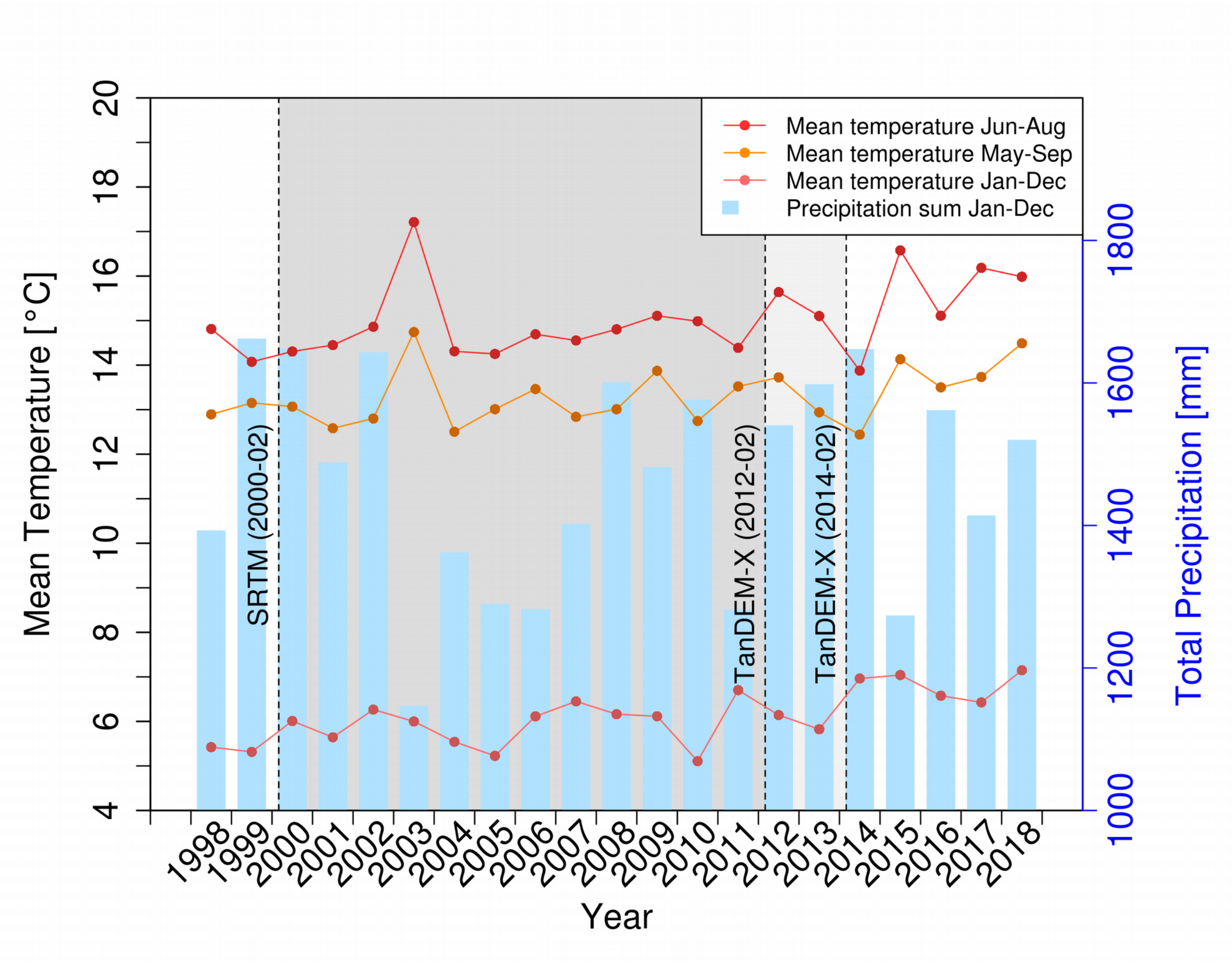


**Supplementary Fig. 6:** Average annual (Jan-Dec) and summer (Jun-Aug) near surface temperatures and total precipitation for 1998-2018 from ERA5 Reanalysis[21] for the greater Alpine region (~5-16°E, 43-48°N). Average temperatures between May and September (orange line) correspond to the approximate glacier ablation period in the Alps. Gray boxes and dotted vertical lines indicate the observation periods and SRTM/TanDEM-X median acquisition dates (on glacierized areas).

**Supplementary Figure 7**

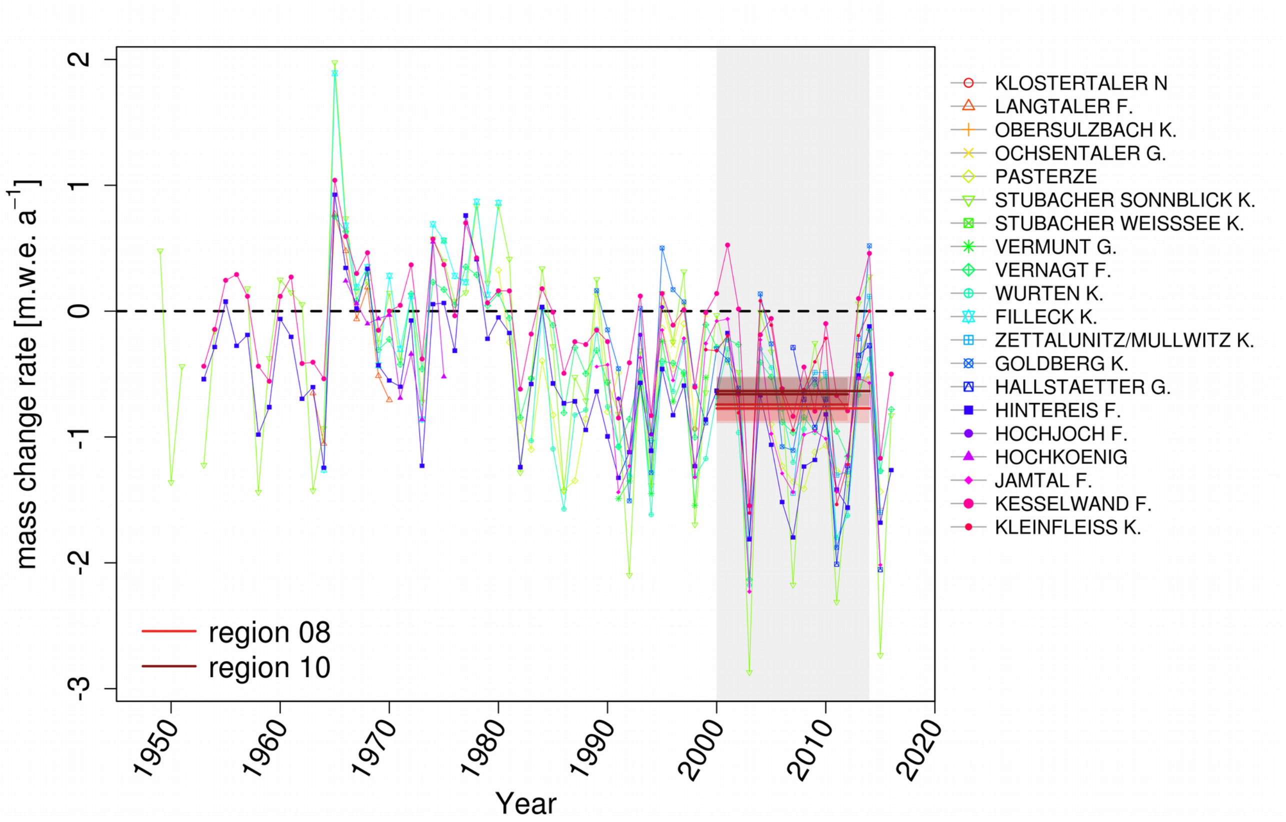


**Supplementary Fig. 7:** Annual glaciological mass change[13] of glaciers in the Austrian Alps in comparison to measurements of region 08 and 10 of this study (observation periods 2000-2012 & 2000-2014). Semitransparent error boxes refer to regional mass change uncertainties (see methods section).

**Supplementary Figure 8**

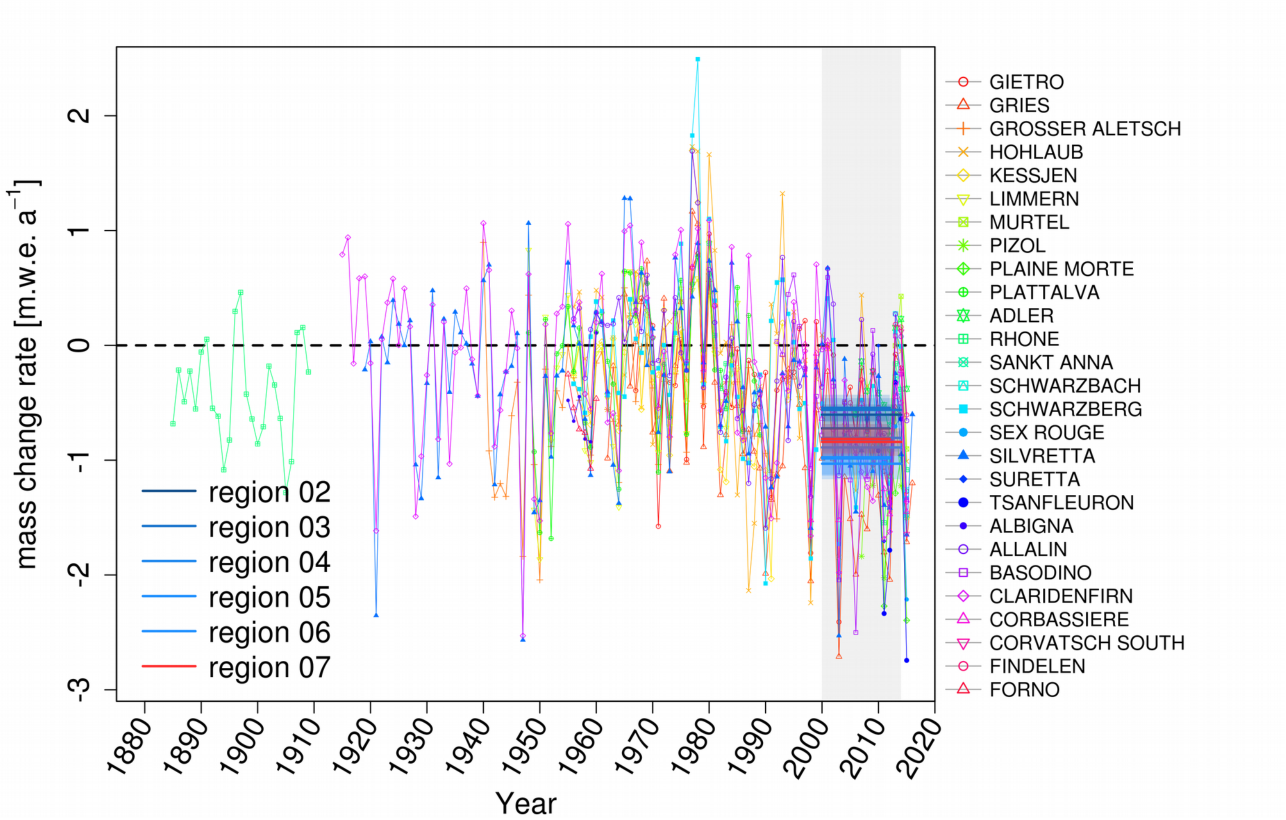


**Supplementary Fig. 8:** Annual glaciological mass change[13] of glaciers in the Swiss Alps in comparison to measurements of region 02-07 of this study (observation periods 2000-2012 & 2000-2014). Semitransparent error boxes refer to regional mass change uncertainties (see methods section).

**Supplementary Figure 9**

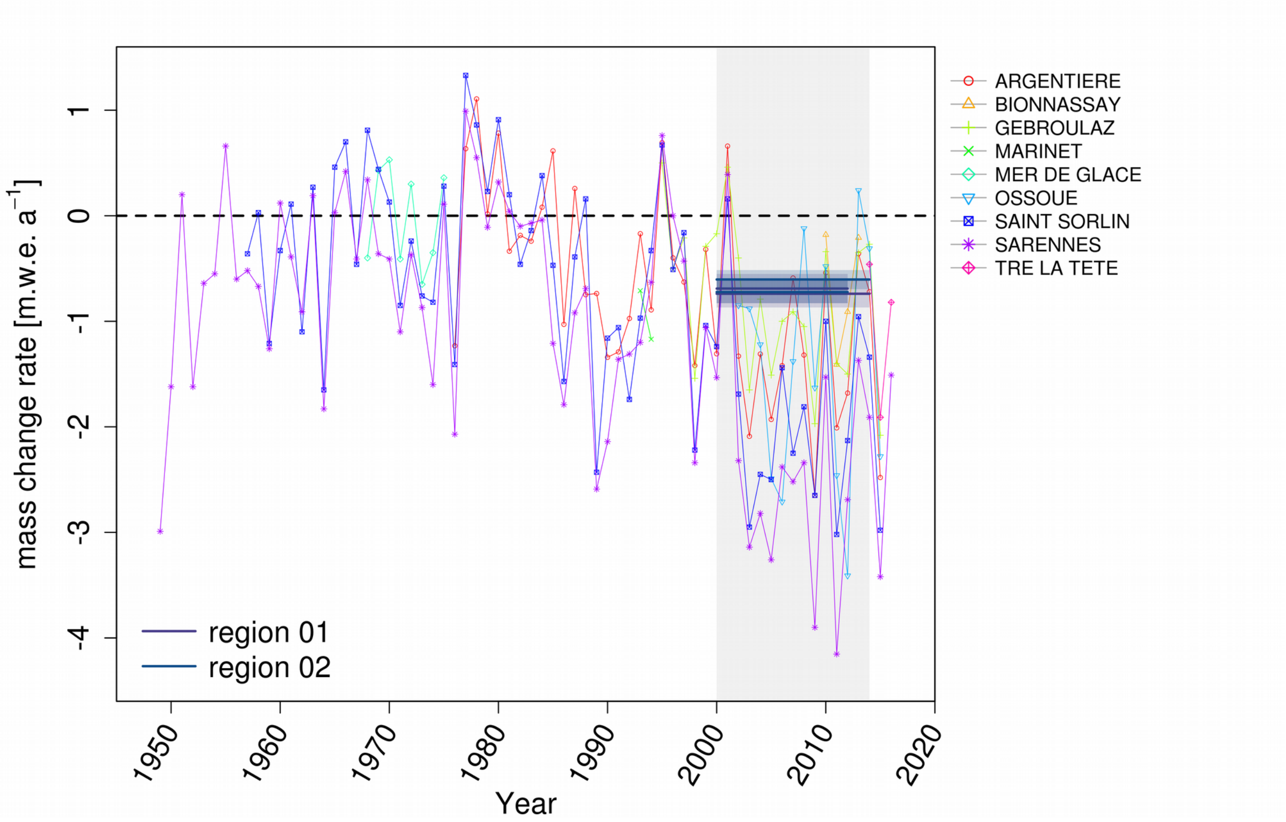


**Supplementary Fig. 9:** Annual glaciological mass change[13] of glaciers in the French Alps in comparison to measurements of region 01 and 02 of this study (observation periods 2000-2012 & 2000-2014). Semitransparent error boxes refer to regional mass change uncertainties (see methods section).

**Supplementary Figure 10**

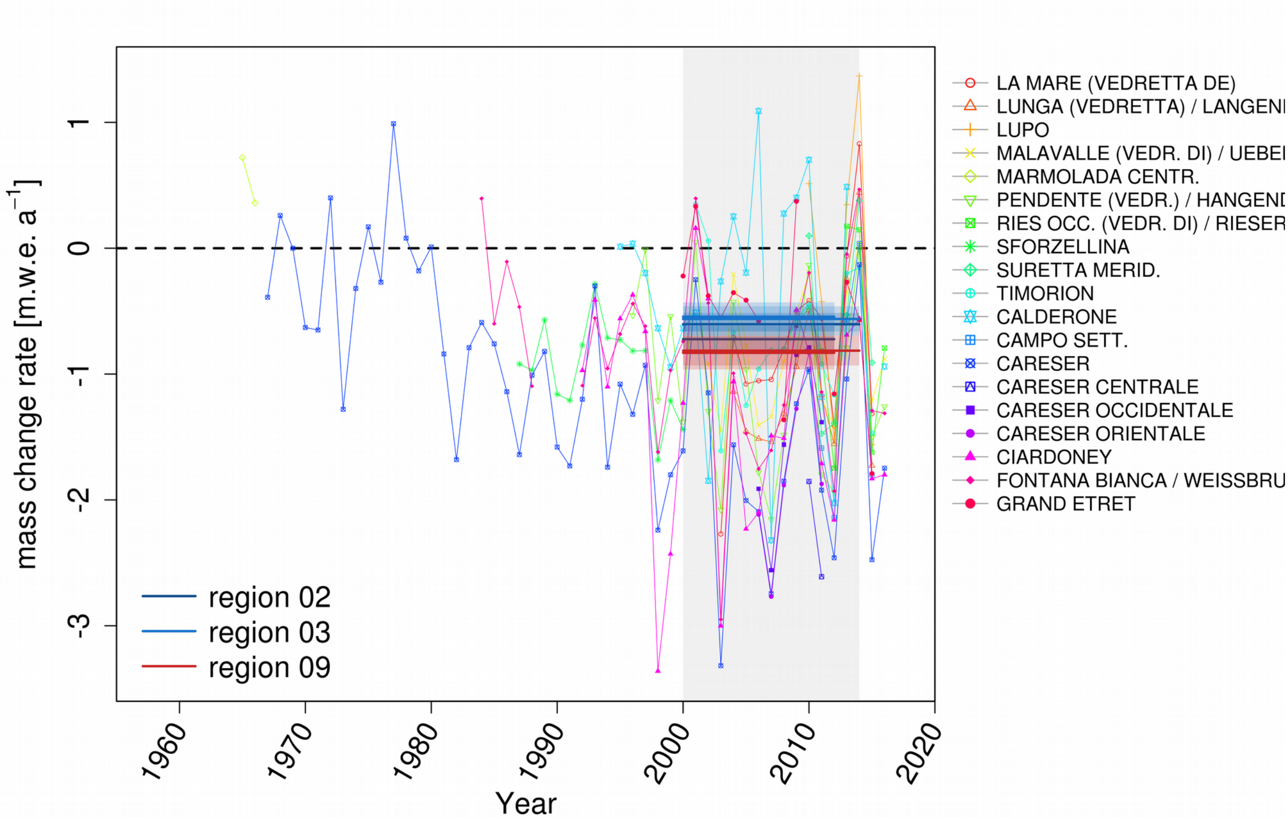


**Supplementary Fig. 10:** Annual glaciological mass change[13] of glaciers in the Italian Alps in comparison to measurements of region 02, 03 and 09 of this study (observation periods 2000-2012 & 2000-2014). Semitransparent error boxes refer to regional mass change uncertainties (see methods section).

## Supplementary Figure 11

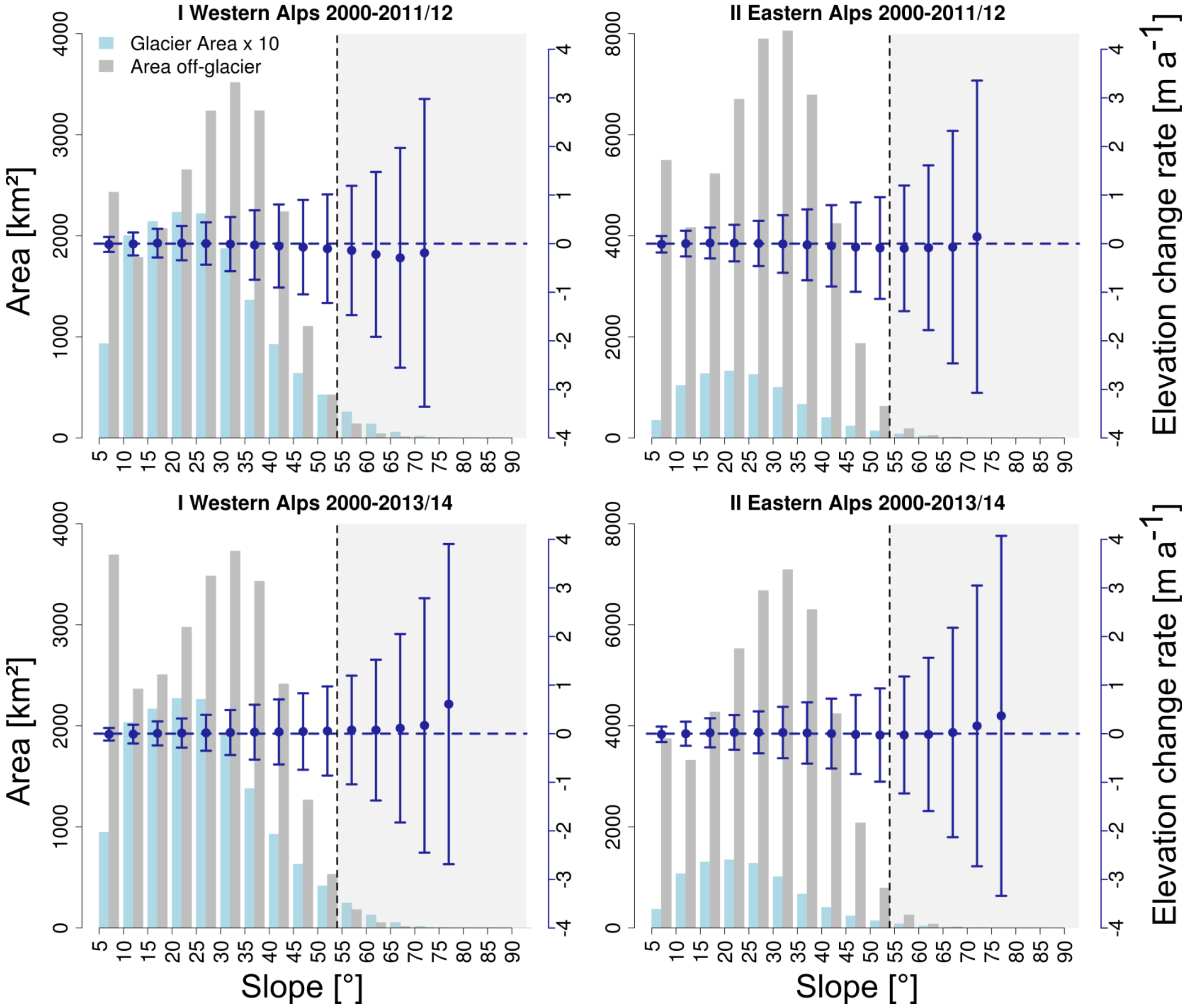


**Supplementary Fig. 11:** Elevation difference vs slope between SRTM and TanDEM-X after corregistration on all non-glacier areas for Western and Eastern Alps and both observation periods. Blue dots and error bars show median elevation change and 1 respective standard deviation, aggregated within 5° slope bins and filtered (2-98% quantile). Gray bars indicate non-glaciated area of each slope bin and blue bars respective glacier area (multiplied by factor 10). Change values above 50° slope are excluded from the analysis (vertical dotted lines at 50°). Change values of slope bins with less than 0.5 km² area are not shown. Note: Different scaling on area axis.

**Supplementary Table 3**

DEM corregistration statistics on non-glacierized areas for each subregion. Total area refers to the extent of the entire subregions. No-ice areas are all areas covered by SRTM and TanDEM-X which are not glacier covered. Standard deviations (SD) of all non-glacier areas were aggregated within 5° slope bins and filtered (2-98% quantile filter). Regional SDs are area-weighted based on the respective glacier areas per slope bin. SD area-w. shows the glacier area-weighted standard deviations of all non-glacier areas less than or equal to 50° and 35° slope.

| *ID* | *Region Name* | *Glacier Area <= 50° [km²]* | *Glacier Area <= 35° [km²]* | *No-ice Area <= 50° [km²] 2000-2012* | *SD area-w. <= 50° [m a⁻¹] 2000-2012* | *No-ice Area <= 50° [km²] 2000-2014* | *SD area-w. <= 50° [m a⁻¹] 2000-2014* | *No-ice Area <= 35° [km²] 2000-2012* | *SD area-w. <= 35° [m a⁻¹] 2000-2012* | *No-ice Area <= 35° [km²] 2000-2014* | *SD area-w. <= 35° [m a⁻¹] 2000-2014* |
|---|---|---|---|---|---|---|---|---|---|---|---|
| 01 | Dauphiné Alps | 83 | 65 | 2978 | 0.460 | 3778 | 0.399 | 2526 | 0.417 | 3273 | 0.350 |
| 02 | Graian Alps | 331 | 283 | 5384 | 0.413 | 5778 | 0.317 | 4623 | 0.374 | 4885 | 0.284 |
| 03 | Pennine Alps | 431 | 381 | 4232 | 0.377 | 3840 | 0.330 | 3483 | 0.344 | 3099 | 0.297 |
| 04 | Bernese Alps | 471 | 413 | 1647 | 0.383 | 1715 | 0.360 | 1375 | 0.345 | 1404 | 0.328 |
| 05 | Glarus Alps | 53 | 48 | 1473 | 0.391 | 1461 | 0.381 | 1262 | 0.351 | 1244 | 0.360 |
| 06 | Lepontine Alps | 54 | 50 | 4222 | 0.357 | 4398 | 0.331 | 3397 | 0.333 | 3472 | 0.312 |
| 07 | Rhaetian Alps West | 146 | 131 | 6614 | 0.374 | 6887 | 0.336 | 5652 | 0.349 | 5749 | 0.309 |
| 08 | Rhaetian Alps East | 219 | 201 | 3989 | 0.346 | 4114 | 0.317 | 3336 | 0.318 | 3350 | 0.295 |
| 09 | Rhaetian Alps South | 124 | 112 | 3323 | 0.323 | 3323 | 0.313 | 2850 | 0.297 | 2834 | 0.285 |
| 10 | Tauern Alps West | 208 | 186 | 5638 | 0.444 | 5584 | 0.394 | 4747 | 0.419 | 4540 | 0.377 |

**Supplementary references**


1. Paul, F., Frey, H. & Le Bris, R. A new glacier inventory for the European Alps from Landsat TM scenes of 2003: challenges and results. Ann. Glaciol. 52, 144–152 (2011).

2. Gardent, M., Rabatel, A., Dedieu, J.-P. & Deline, P. Multitemporal glacier inventory of the French Alps from the late 1960s to the late 2000s. Global and Planetary Change 120, 24–37 (2014).

3. Paul, F., Kääb, A., Maisch, M. & Haeberli, W. The new Swiss Glacier Inventory 2000. in The Swiss Glaciers 2001/2002 and 2002/2003. 51–58 (Herren, E R [et al.]).

4. Bauder, A., Funk, M. & Huss, M. Ice-volume changes of selected glaciers in the Swiss Alps since the end of the 19th century. Ann. Glaciol. 46, 145–149 (2007).

5. Fischer, M., Huss, M., Barboux, C. & Hoelzle, M. The New Swiss Glacier Inventory SGI2010: Relevance of Using High-Resolution Source Data in Areas Dominated by Very Small Glaciers. Arctic, Antarctic, and Alpine Research 46, 933–945 (2014).

6. Knoll, C. & Kerschner, H. A glacier inventory for South Tyrol, Italy, based on airborne laser-scanner data. Ann. Glaciol. 50, 46–52 (2009).

7. Diolaiuti, G. A., Bocchiola, D., Vagliasindi, M., D’Agata, C. & Smiraglia, C. The 1975–2005 glacier changes in Aosta Valley (Italy) and the relations with climate evolution. Progress in Physical Geography: Earth and Environment 36, 764–785 (2012).

8. Carturan, L. et al. Area and volume loss of the glaciers in the Ortles-Cevedale group (Eastern Italian Alps): controls and imbalance of the remaining glaciers. The Cryosphere 7, 1339–1359 (2013).

9. Lambrecht, A. & Kuhn, M. Glacier changes in the Austrian Alps during the last three decades, derived from the new Austrian glacier inventory. Ann. Glaciol. 46, 177–184 (2007).

10. Abermann, J., Lambrecht, A., Fischer, A. & Kuhn, M. Quantifying changes and trends in glacier area and volume in the Austrian O¨ tztal Alps (1969-1997-2006). The Cryosphere 11 (2009).

11. Buckel, J., Otto, J. C., Prasicek, G. & Keuschnig, M. Glacial lakes in Austria - Distribution and formation since the Little Ice Age. Global and Planetary Change 164, 39–51 (2018).

12 Fischer, A., Seiser, B., Stocker Waldhuber, M., Mitterer, C. & Abermann, J. Tracing glacier changes in Austria from the Little Ice Age to the present using a lidar-based high-resolution glacier inventory in Austria. The Cryosphere 9, 753–766 (2015).

13 WGMS 2018. Fluctuations of Glaciers Database. World Glacier Monitoring, Zurich, Switzerland. (2018).

14 Paul, F. & Haeberli, W. Spatial variability of glacier elevation changes in the Swiss Alps obtained from two digital elevation models. Geophys. Res. Lett. 35, L21502 (2008).

15 Kropáček, J., Neckel, N. & Bauder, A. Estimation of Mass Balance of the Grosser Aletschgletscher, Swiss Alps, from ICESat Laser Altimetry Data and Digital Elevation Models. Remote Sensing 6, 5614–5632 (2014).

16 Fischer, M., Huss, M. & Hoelzle, M. Surface elevation and mass changes of all Swiss glaciers 1980–2010. The Cryosphere 9, 525–540 (2015).

17 Zemp, M., Haeberli, W., Hoelzle, M. & Paul, F. Alpine glaciers to disappear within decades? Geophys. Res. Lett. 33, L13504 (2006).

18 Huss, M. Extrapolating glacier mass balance to the mountain-range scale: the European Alps 1900–2100. The Cryosphere 6, 713–727 (2012).

19 Gardner, A. S. et al. A Reconciled Estimate of Glacier Contributions to Sea Level Rise: 2003 to 2009. Science 340, 852–857 (2013).

20 Zemp, M. et al. Global glacier mass changes and their contributions to sea-level rise from 1961 to 2016. Nature 568, 382–386 (2019).

21 Copernicus Climate Change Service (C3S) (2017): ERA5: Fifth generation of ECMWF atmospheric reanalyses of the global climate. Copernicus Climate Change Service Climate Data Store (CDS), *17.12.2019*.
https://cds.climate.copernicus.eu/cdsapp#!/home
